# Prediction of Gait Kinematics and Kinetics: A Systematic Review of EMG and EEG Signal Use and Their Contribution to Prediction Accuracy

**DOI:** 10.3390/bioengineering10101162

**Published:** 2023-10-04

**Authors:** Nissrin Amrani El Yaakoubi, Caitlin McDonald, Olive Lennon

**Affiliations:** School of Public Health, Physiotherapy and Sports Science, University College Dublin, D04 V1W8 Dublin, Ireland; nissrin.amranielyaakoubi@ucdconnect.ie (N.A.E.Y.);

**Keywords:** prediction, gait analysis, electroencephalograms (EEGs), electromyograms (EMGs), kinematics, kinetics, joint angles, joint moments, joint torque, neural network

## Abstract

Human-machine interfaces hold promise in enhancing rehabilitation by predicting and responding to subjects’ movement intent. In gait rehabilitation, neural network architectures utilize lower-limb muscle and brain activity to predict continuous kinematics and kinetics during stepping and walking. This systematic review, spanning five databases, assessed 16 papers meeting inclusion criteria. Studies predicted lower-limb kinematics and kinetics using electroencephalograms (EEGs), electromyograms (EMGs), or a combination with kinematic data and anthropological parameters. Long short-term memory (LSTM) and convolutional neural network (CNN) tools demonstrated highest accuracies. EEG focused on joint angles, while EMG predicted moments and torque joints. Useful EEG electrode locations included C3, C4, Cz, P3, F4, and F8. Vastus Lateralis, Rectus Femoris, and Gastrocnemius were the most commonly accessed muscles for kinematic and kinetic prediction using EMGs. No studies combining EEGs and EMGs to predict lower-limb kinematics and kinetics during stepping or walking were found, suggesting a potential avenue for future development in this technology.

## 1. Introduction

The activity of walking or stepping is complex, involving the simultaneous control of multiple physiological systems. This whole-body motion is thus an adaptive strategy that maintains postural control of the body’s centre of mass while executing volitional movement [1]. The conservation of the body’s centre of mass and the execution of effective motor tasks requires the integration and modulation of sensory signals from visual, somatosensory, and vestibular sources in the nervous system and the interconnectivity between motor structures in the brain (e.g., motor, pre-motor, supplementary motor, and associative cortices alongside the thalamus, basal ganglia, and cerebellum), to generate appropriate, adaptive movements [2]. Neurological disorders impair the interconnection of systems, making motor control movement more difficult, thus affecting the lower limbs leading to reduced mobility and life quality [3]. Effective therapies to help retore functional walking need to target the neurophysiological basis of gait [4].

Neurological rehabilitation, virtual-reality, gaming, and other paradigms employing human–machine interfaces (HMIs) are continually developing for movement restoration [5,6]. Brain–computer interface (BCI) technology, a component of HMIs, refs. [7,8] records, analyses, and converts brain signals into electrical commands for another device. While different BCIs exist, they are normally used for defining the behaviour of an external device, as is the focus in this study [9]. BCI technology has grown exponentially over the last few decades in rehabilitation compared to other tools due to its capacity to induce positive neuroplasticity and preserve and/or improve muscle strength [10,11]. It has been proposed that the BCIs can nourish damaged neurological connections between the brain and muscle [12,13]. Different neurophysiological signals (electrocorticography (ECoG), EEG, magnetoencephalography (MEG), functional magnetic resonance imaging (fMRI), among others) can be used. When a BCI employs only central neurophysiological signals, this system is purely BCI. However, brain signals can be complemented with other physiological data in systems called dependent BCIs [14]. Both systems have their pros and cons. While dependent BCIs increase the percentage of noise fed to the system and the computational resources required, the multiple biosignal types can provide helpful information from different biological systems, making them more robust to different movements and situations. There is currently no clear evidence which system is superior; consequently, both types of BCIs will be explored in this review with “BCI” used to denote both types of systems. 

Current BCI research and development is largely focused on the advancement of upper-limb BCI technology where numerous studies report BCI efficacy when compared to conventional therapies in affecting immediate and long-term improvements in upper-limb motor function [3,12,15]. Lower-limb BCI lags behind where the focus remains on designing BCIs to achieve the same efficiency and patient impact as upper-limb technology. More directed scientific efforts are required, as lower-limb BCIs can involve differing movements from simple (e.g., flexion and extension of joints), to more complex multi-joint activities such as sit-to-stand, stepping forward, backwards, stepping up, climbing stairs, ramp negotiation, walking overground, over treadmill, etc. Each activity and each platform over which the activity is conducted generate different neurological and musculoskeletal patterns. In this current review, we consider only walking and stepping activity as these are an essential functional, complex movement [16,17]. 

BCI systems are composed of six key functional blocks [18]: (i) signal acquisition representing the devices used to collect neural data, (ii) signal pre-processing defining which mathematical tools will be applied to the neural signals to eliminate information that is not relevant for the prediction, (iii) feature extraction defining how data will be described and which variables will be fed to the prediction system, (iv) prediction where the input data is translated into an output based on algorithms such as statistical models, regression methods, tree algorithms, and neural networks. The translation process can be further grouped based on the output type of discrete (limited to several labels) or continuous where the output can have any value. This translation process can be further grouped based on the output type of discrete (limited to several labels) or continuous (our focus) where the output can have any value. The final functional blocks (v) control, and (vi) feedback use output generated from the prediction block as additional input of the prediction block for future operations. The main goal of these final blocks is to reduce system error over time. 

This systematic review of the literature aims to provide a comprehensive knowledge base of current state-of-the-art data that best predict continuous lower-limb kinematics and kinetics during stepping/walking. Moreover, consolidating all published data will highlight the existing scientific gaps and future work required to expand and improve BCI technology in lower-limb rehabilitation. The objectives of this review are to answer the following questions: which brain regions and lower-limb muscles are most commonly used, and which are the most informative for accurately predicting lower-limb kinematics and kinetics during gait or stepping activity in healthy individuals and in those with central neurological disorders? Which tools are currently used to predict lower-limb kinematics and kinetics when fed with EEGs and/or electromyograms (EMGs)? Which systems provide the greatest accuracy?

## 2. Materials and Methods

This systematic review was conducted in line with best practice guidelines [19] adhering to the following iterative steps: (i) definition of the research question informed by the PICO acronym, (ii) identification of the electronic databases that will be searched and development of a comprehensive wide-reaching search strategy, (iii) collation of the citations identified from each database into EndNote reference manager software, (iv) elimination of duplicate papers and transfer of the remaining papers to Covidence software for all subsequent screening stages by two independent reviewers (CMD and NAEY), (v) title screening of the remaining papers, (vi) independent abstract screening, (vii) full-text paper screening, (viii) quality assessment of the papers selected for the review, again by independent reviewers (CMD and NAEY), and (ix) data extraction. The procedure followed to accomplish this systematic review have been published by PROSPERO, ID: CRD42021289711.

Research manuscripts reporting large datasets that are deposited in a publicly available database should specify where the data have been deposited and provide the relevant accession numbers. If the accession numbers have not yet been obtained at the time of submission, please state that they will be provided during review. They must be provided prior to publication.

Interventionary studies involving animals or humans, and other studies that require ethical approval, must list the authority that provided approval and the corresponding ethical approval code.

### 2.1. Inclusion and Exclusion Criteria

Inclusion criteria comprise any scientific paper providing participant data that predict lower-limb kinematics and/or kinetics (joint moment and torque) during flat overground gait or stepping performance using recordings of brain activity (EEG signals) and lower-limb muscle activity (EMG signals); studies examining adult participants (over 18 years old) who are reported as healthy or as having central, named neurological pathology.

Studies that report data from animals, human participants younger than 18 years of age, participants with a diagnosed pathology other than a central neurological condition including amputations, or studies that did not include 3D kinematic data co-registered with either EEG signals and/or EMG signals were excluded. Studies where the walking activity was performed on a treadmill, where electrical or magnetic stimulation or medication was provided during or preceding the walking or stepping activity, where participants were required to complete a cognitive task during the activity, or where perturbations were applied during walking were excluded from this review because of potential activation of different brain regions and/or lower-limb muscles in comparison to the default state of human walking or stepping [20,21,22]. Studies recording only kinetic data related to the stepping activity were excluded, with the exception of those reporting angular velocities, accelerations, and torques. These were included as they provide information about the movement of the limb(s) in space and are directly correlated with kinematic data. Other kinetic data, such as ground force (GRF), were excluded [23]. Studies reporting prediction systems that are not continuous were also excluded as the participant is not actively engaged during movement, an aspect of high importance for rehabilitation [4].

### 2.2. Study Design

Five electronic databases were searched for relevant materials: PubMed and Embase, oriented to biomedical sciences, CINHAL focused on biomedicine and healthcare from a biological perspective, and Scopus and Web of Science for engineering studies related to biomedicine. The key search terms applied across all databases were EMG, EEG, brain–machine interface/brain–computer interface, locomotion, stepping, kinematics, ERD/ERS/ERSP, and MTP. The eight search strings were combined using Boolean operator as follows (1 OR 2 OR 3 OR 4) AND (5) AND (6 OR 7 OR 8) taking consideration of the inclusion and exclusion criteria. The Boolean operators, controlled vocabulary terms (e.g., MESH in PubMed), and punctuations were modified to the unique search rules for each database. The rationale for structuring the key terms and employing Boolean operations in this investigation lies in the aim of identifying studies that utilized the subsequent data sources: Electroencephalography (EEG), Electromyography (EMG), derived variables such as Event-Related Synchronization/Desynchronization (ERS/ERD/ERSP), which are outcomes of computations performed on EEG data, gathered during stepping, or employed Brain–Computer Interface (BCI) devices during stepping. Furthermore, these studies must have assessed kinematic parameters, or alternatively, forecasted, or scrutinized motion. We deliberately maintained a broader search string than the specific research inquiry to mitigate the risk of overlooking potentially relevant publications within the context of this systematic review. Variations and synonyms of these keywords formulated individual search strings based on the PubMed search rules, included as an example in Table 1. In the context of this study, the notation ‘#’ denotes “sentence number” whereas the symbol ‘*’ has been incorporated into the search process to identify derivatives of root words. For example, it is employed to find derivatives of terms like “electroencephalog*” yielding results such as “encephalography” and “encephalograms”.

The resulting scientific papers identified in each database were exported to EndNote, where duplicate studies were identified and eliminated. The remaining scientific papers were first screened by title to eliminate those which were clearly not related to the question at hand, the remaining papers were then screened independently by the two reviewers (NAEY and CMD) by abstract and finally by reading the full texts, identifying which studies met the inclusion criteria and eliminating those with clear exclusion criteria. Disagreements between reviewers were addressed through discussion, re-examination of the aims and inclusion criteria and with a third party (OL), where required, to reach consensus.

### 2.3. Quality Assessment Tool

The quality of the included research papers was assessed by two independent reviewers using the Effective Public Health Practice Project (EPHPP) tool to identify biases in the reported research that may affect review conclusions. The EPHPP tool was chosen as it demonstrates acceptable inter-rater reliability, the objectivity of their template questionnaire and its versatility across multiple research methodologies [24]. In this study, each paper underwent assessment from eight distinct perspectives employing EPHPP tool. Each assessment was evaluated as weak, moderate, or high quality. All studies included in this systematic review have been classified as “cross-sectional studies”—meaning “Confounders”, “Blinding”, and “Withdrawal sections” were rated as not applicable. Subsequently, an overall quality rating was assigned to each study following the EPHPP methodology. These eight perspectives pertain to:

1. Selection bias: Examining potential biases in the selection process of the study participants.
2. Study design: Evaluating the robustness and appropriateness of the chosen research design.
3. Confounders: Analysing potential variables that may impact the study’s conclusions.
4. Blindings: Evaluating whether subjects are aware of the study design in a manner that could influence the data they contribute.
5. Data collection method: Scrutinizing the accuracy and appropriateness of the instruments employed during data collection for the study.

Additionally, the assessment encompassed an evaluation of withdrawals and drop-outs, specifically addressing any challenges or issues faced by researchers during the course of the study.

### 2.4. Data Extraction Strategy

A proforma was developed to aid uniformity during data extraction by two independent reviewers. Information was extracted from each included paper under the following headings.

- Participants characteristics (sex, age, healthy subject, or with a central neurological disorder, weight, height)
- Test protocol
- Data collected (input and output)
- Pre-processing pipeline applied to input and output signals
- Prediction tool utilised
- Accuracy of prediction

Firstly, a description of participants and protocols was collated to obtain an overview of the physiological characteristics of the participants and protocols applied for walking or stepping activity (with or without speed control). Secondly, registration of the data collected to feed the prediction system was collated, and the associated pre-processing performed was extracted. Thirdly, a compendium of prediction systems and their performance was developed and classified into four groups as guided by Caldas et al. [25] as: neural networks, regression methods, statistical models, and tree algorithms.

Results were reported narratively for the number and description of included studies and participants involved. A detailed description of the protocol, analysis, and conclusions drawn from each study was then collated, categorized as studies which performed kinematic or kinetic predictions using only brain activity (EEGs), only lower-limb muscle(s) activity (EMGs) and studies that performed kinematic or kinetic prediction with a combination of lower-limb muscle activity and/or other parameters. For EEG-based BCIs, the most informative brain zone(s) related to the lower-limb kinematics and kinetics was considered, while for EMGs and combined data types, the lower-limb muscles which were commonly utilised across studies and those related to the highest prediction accuracy were extracted.

## 3. Results

**Figure 1:**
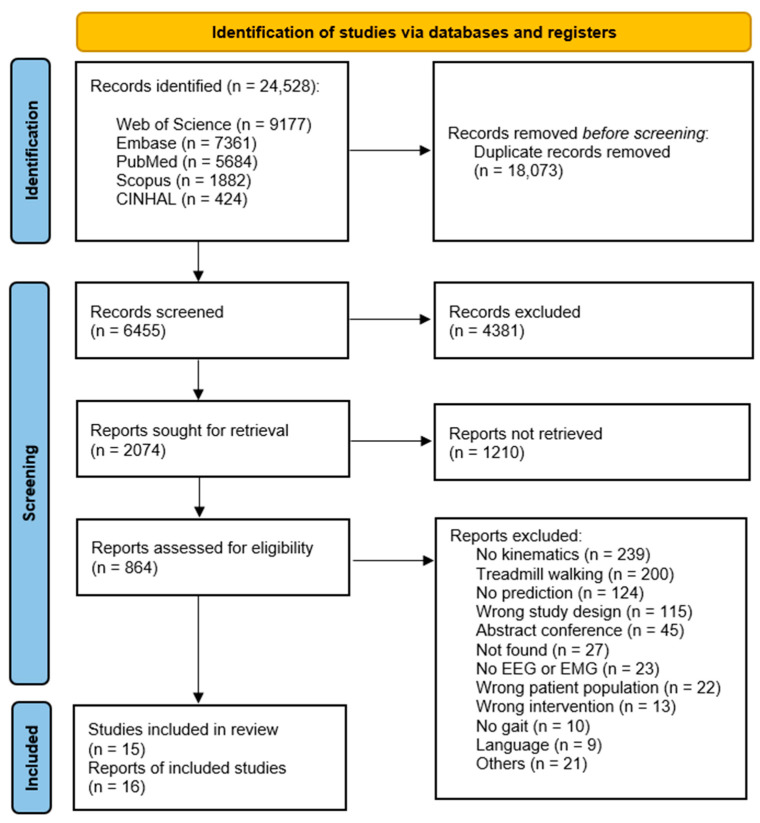
PRISMA flow diagram [19].

**Figure 2:**
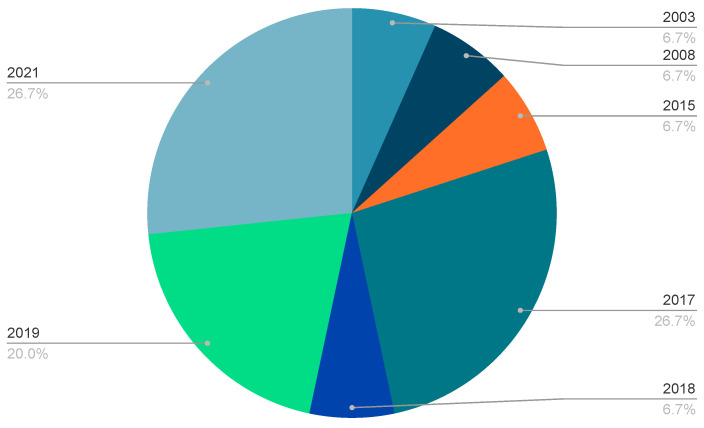
Distribution of the publication dates of included studies.

As identified in the flow diagram (Figure 1), a total number of 16 studies were included in the review following all screening stages. Only 15 studies are discussed in detail in the results as one study [26] did not provide sufficient detail, for example in relation to their protocol, participant characteristics, pre-processing procedures. Multiple attempts to contact the authors for further information failed. The two main reasons for the exclusion of studies related to the absence of 3D kinematic data or where gait was recorded during treadmill walking (detailed in Figure 1). With no limitation applied in the search strategy related to date of publication, the papers included ranged from 2003 up to 2021 (Figure 2), with the median publishing median date 2018. Across all included studies, participants comprised a total of 121 healthy subjects and six individuals with hemiparesis in the chronic phase post-stroke. Prediction of lower-limb kinematics and kinetics in the included studies utilised models deploying EEG data (N = 2), EMG data (N = 8) and EMG and additional kinematic data (N = 5). No study identified combined EEG and EMG biosignals.

**Figure 3:**
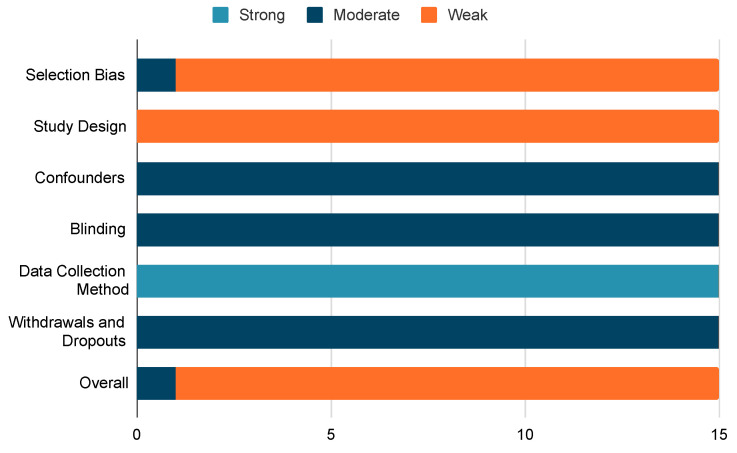
Summary of quality assessment results applied to the included studies.

Of the 15 studies reported in this review, 14 were considered to be of weak quality, when rated by the EPHPP tool. Only one study [27] was rated by reviewers to be higher (moderate quality). The primary risks of bias identified in the included studies related to the absence of a targeted procedure for participant recruitment and the cross-sectional nature of many of the studies, as summarised in Figure 3.

Studies were classified into three categories based on input fed to the prediction systems: (i) studies that employed only EEG to predict lower-limb kinematics and kinetics, (ii) studies that employed only EMG to predict lower-limb kinematics and kinematics, and (iii) studies that employed EMG and other variables to predict lower-limb kinematics and kinetics. From each category, a series of conclusions after the analysis of paper is displayed in Table 2. A detailed description of the studies can be found in the following subsections.

### 3.1. Lower-Limb, Kinematic, and Kinetic Prediction with EEGs

Two of the included studies used only EEG data for kinematic and kinetic prediction, summarised in Table 3. Mercado et al. [28] predicted hip and knee torques offline in 20 healthy subjects when stepping forward and stepping up using a multi-layer perceptron (MLP) neural network. This MLP was fed with EEGs in the time-domain from central and peripheral zones of the brain using 19 electrode sites (FP1, FP2, F7, F8, F3, F4, T3, T4, C3, C4, T5, T6, P3, P4, O1, O2, FZ, CZ, and PZ). Joint torques were calculated based on the Denavit–Hartenberg notation and the Euler–Lagrange approach, where the joint angles were computed by processing video recordings of subjects during the movements. This study established that stepping kinetics (hip, knee torques) can be decoded offline from time domain EEGs. The performance of the decoder, evaluated using the coefficient of determination, correlation coefficient, and signal-to-noise ratio, found greatest accuracy in predicting hip torque (right hip outperforming left hip). The prediction of a step forward preformed best during the first test session, where C3 and C4 electrodes contributed relevant information.

During the second session C3, CZ, and P3 electrodes provided the most meaningful information. When stepping up, the best predictive performance was during the later (third and fourth) test sessions where F4, C4 and F4, and F8 electrodes contributed the highest relevant information to the decoder.

In the second EEG-based prediction study, Contreras-Vidal et al. [27] predicted hip, knee, and ankle angle joints offline in five individuals in the chronic phase of stroke during walking with powered exoskeleton assistance. One of the participants could not finish the experiment. A 10th order unscented Kalmar decoder fed with delta band frequencies (0.1 to 3 Hz) from 48 EEG channels organised on the 10–20 standard system was employed. Interventionary studies involving animals or humans and other studies requiring ethical approval must list the authority that provided approval and the corresponding ethical approval code. Unlike the previously EEG-based study, which used a neural network as a prediction tool, Contreral-Vidal et al. [27] used a statistical model. Peripheral EEG channels (FP1, FP2, AF7, AF8, F7, F8, FT7, FT8, FT10, T7, T8, TP7, TP8, TP10, PO9, PO10) were excluded due to their susceptibility to recording muscle activity [29,30]. Root mean squared error (RMSE) for prediction accuracy of hip, knee, and ankle joint angles, measured by the H2 exoskeleton, were calculated demonstrating decoding from frequency domain EEG following stroke is feasible. However, the decoder performance accuracy was dependent on the gait parameters (total steps, steps per minute, and walking speed) of the individual and improved with multiple gait sessions.

**Figure 4:**
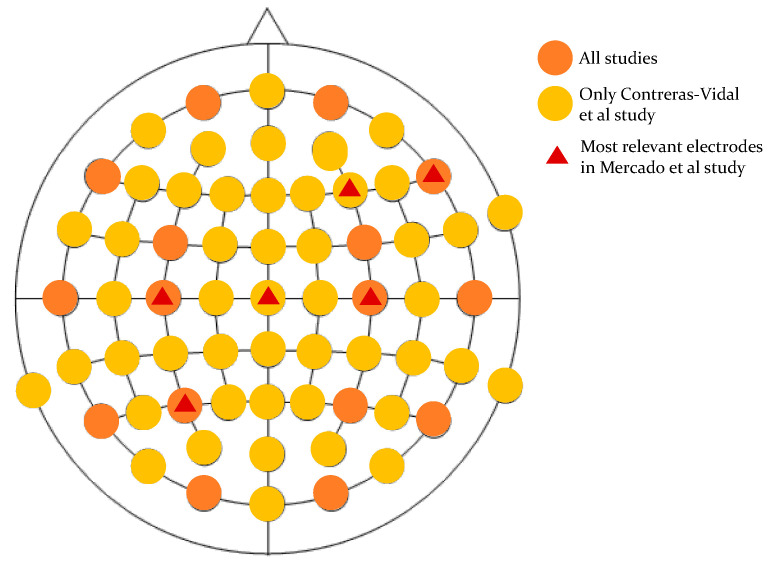
Conjunction and disjunction of EEG sites used in EEG-based kinematic and kinetic prediction studies [22,23].

Figure 4 represents the electrode positions used by EEG studies to predict lower-limb kinematic and kinetic data. Orange circles represent the location of the electrodes commonly used by Mercado et al. [28] and Contreras-Vidal et al. [27] for the EEG acquisition, yellow circles refer to the location of the electrodes included only in the Contreras-Vidal et al. study, while the red triangles mark the electrodes that have been defined as valuable for a good accuracy prediction by the Mercado study. While Contreras-Vidal et al. [27] did not evaluate the contribution of each electrode to the overall prediction accuracy, the six electrodes identified by Mercado et al. [28] as having the highest relevance for good accuracy prediction of lower-limb torques had central locations (C3, C4, Cz, P3, F4, F8).

Even though the major differences between these two EEG-based studies exist with respect to pre-processing pipelines, the mathematical prediction tools and performance metrics are as summarized in Table 3.

### 3.2. Lower-Limb, Kinematic, and Kinetic Prediction with EMGs

As detailed in Table 4, eight studies included in this review predicted lower-limb kinematic data using muscle activity as input to their prediction systems [31,32,33,34,35,36,37,38]. All studies involved healthy subjects with detailed demographic information provided in six studies and minimal information provided in three [31,34,38]. Where identified, 39 male participants and 6 female participants, with an average age of 30 years (no subject >50 years) and with weight ranging from 60 to 80 kg were included across all studies.

All protocols instructed participants to walk overground without assistance. Two studies included additional activities such as stair descent/ascent, ramp descent/ascent [31], and sitting and standing [34]. Only one study [32] controlled participants’ gait speed. Five papers predicted knee angle joints using unilaterally muscle activity [34,35,36,37,38], four of which measured the left limb [34,35,37,38]. One study, by Li et al. [36], did not identify measurement laterality. One study predicted knee and ankle joint angles [31], and two studies predicted hip, knee, and ankle joint angles from only one limb [32,33] using the right and left limb.

Different prediction tools were used across studies: an unscented Kalman filter [31], backpropagation (BP) neural network [32], dynamic recurrent neural network (DRNN) [33], random forest (RF) [36], long-short term memory (LSTM), and convolutional neural network (CNN) [34,35,37].

The majority (four out of six) of prediction tools reported in these EMG-based studies were neural networks (BP, DRNN, CNN, and LSTM), with no consistency in the neural network(s) utilised apparent. No regression prediction models defining the relationship between output and input by a linear regression model was identified using EMG data.

To evaluate predictive performance, two studies used root mean square error (RMSE) and Pearson’s correlation coefficient (CC) as their evaluation metrics [35,38], four studies used only RMSE [32,35,36,37]. One study [31] employed only CC and one final study [34] evaluated the performance of the prediction system by MAE. 

**Figure 5:**
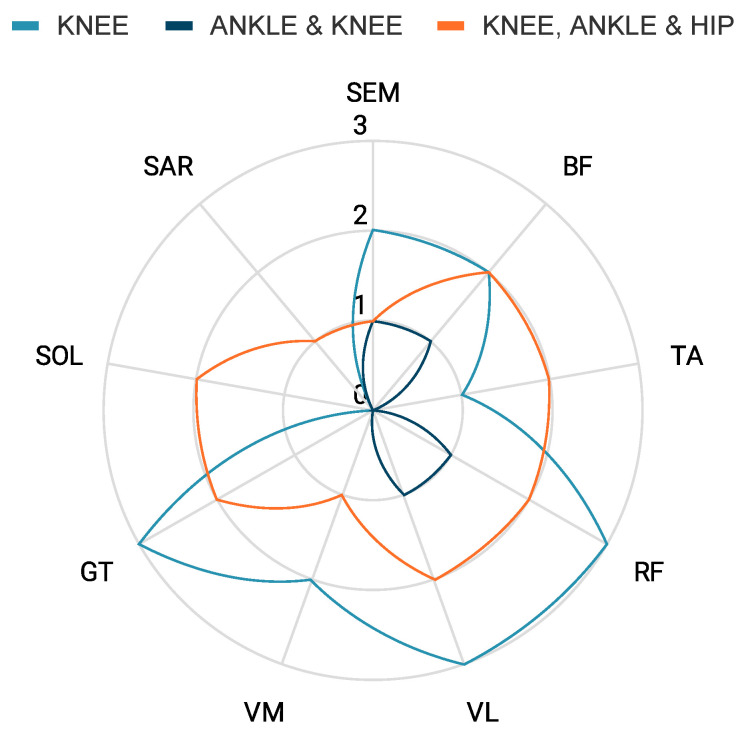
Representation of the muscle activity used for each lower-limb kinematic prediction in studies with only EMGs as the input.

Figure 5 depicts the muscle groups generating the EMG data used to feed the prediction systems and the continuous outcomes generated from included studies. In this figure, each circle represents number of papers, while the radiating lines from the center denote different muscles. Each data point signifies how many papers have incorporated each respective muscle in their research. The lower-limb muscles where the EMG was most recorded across studies were Rectus Femoris (RF, N = 6), and Vastus Femoris (VF, N = 6), followed by Biceps Femoris (BF, N = 5) and the Gastrocnemius muscle (GT, N = 5). Notably, the most commonly used muscles to predict knee angle were GT (N = 3), Vastus Lateralis (VL, N = 3), and RF (N = 3). The predictions with the highest identified accuracies were by Gautam et al. [34] and Jia et al. [35] with a mean absolute error (MAE) of 8.1% and RMSE of 0.464° reported, respectively. Both studies predicted only knee angles using left lower-limb muscle activity from Vastus Medialis (VM), Semitendinosus (SEM), BF, and RF muscles fed to CNN and LSTM [34] and RF, VL, and GT activity fed to LSTM-only [35] neural networks.

Five out of the eight studies drew conclusions in relation to kinematic/kinetic prediction that warrant future exploration. Chen et al. [32] predicted hip, knee, and ankle joints employing back propagation and principal component analysis (PCA) evaluated with RMSE. The results showed the BP neural network outperformed the PCA method in extracting optimal feature vectors for multichannel surface EMG to capture the shape of the angle curve. This resulted in lower errors of angle estimation for each joint by the DBN method compared to PCA, while cross-correlation coefficients were higher. Cheron et al. [33] predicted thigh, shank, and foot elevation angles, their angular velocity and acceleration employing DRNN. Evaluation using RMSE identified DRNN is best at predicting angular velocity in comparison to angles and acceleration due to its low error rate, rapid converge, and avoidance of bifurcation.

Jia et al. [35] studied the performance of traditional LSTM, their adopted LSTM model, and traditional RNN for predicting knee joint trajectory. The results revealed that adopted LSTM (where angle joints were not fed to the same LSTM block as the activity muscles to extract more discriminative features) more closely predicted the actual trajectory than traditional LSTM, and demonstrated better smoothing capability than RNN. Thus, LSTM neural networks with feature-level fusion (i) can accurately learn various motion characteristic data of lower limbs, which is promising for modelling gait, (ii) the average value of the correlation coefficient generated is larger than the LSTM or RNN models, indicating better regression performance, and (iii) has good adaptability and universality for different subjects. Liu et al. [37] examined estimated knee joint angle using three models: data-based CNN, feature-based CNN, and BPNN, where errors between the experimental angle and model estimated angle were larger in data points near the second peak knee flexion (data-based CNN > BPNN > feature-based CNN), while errors in the other data points were similar. Li et al. [36], in comparing knee angle prediction performance between random forest (RF) and back propagation (BP) employing PCA, identified RFPCA as closer and more robust to the experimental measurements (EMs) than BPPCA. However, as the sample size increased the estimations of BPPCA and RFPCA became closer to the EM, although FRPCA was deemed less time-consuming.

### 3.3. Lower-Limb, Kinematic, and Kinetic Prediction with EMG and Additional Data

As detailed in Table 5, five studies included in this paper predicted lower-limb kinematic and kinetic data by using muscle activity and joint(s) angle as input to the prediction system [39,40,41,42,43]. All studies involved healthy subjects (N = 51, 28 males; 23 females; mean age 30 years, mean height 170 cm, and weight ranging between 60 kg and 80 kg). All participants were instructed to walk overground without assistance or exoskeleton use. Two studies requested participants to walk at their natural speed [42,43], two studies [40,41] requested gait at natural speed and at different speeds. One study [39] controlled participants’ speed during the experimental protocol. All studies measured activity in lower-limb muscles bilaterally, with one exception [40]. Data inputted to predict kinematic and kinetic variables were composed of two types: muscle activity and other physiological information such as demographics, anthropometrics, joints angles, among others, as detailed in Table 5.

**Figure 6:**
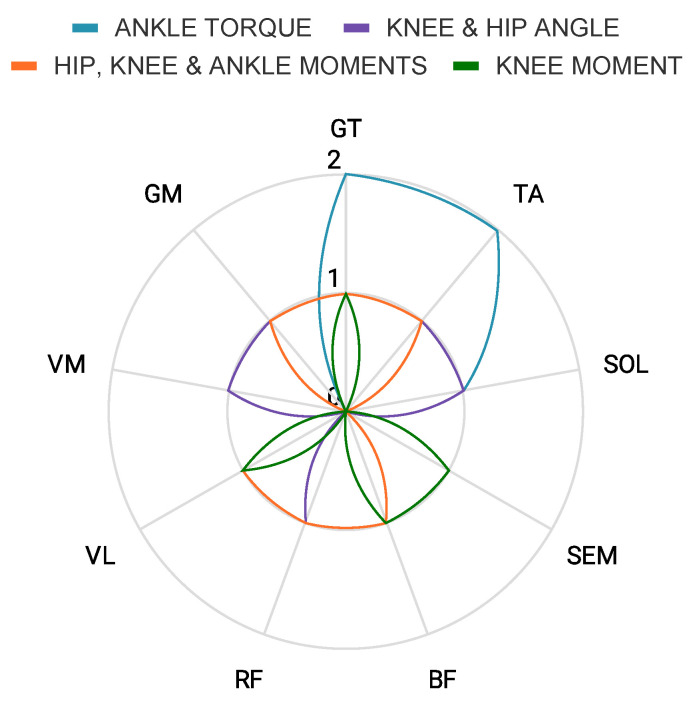
Representation of the muscle activity used for each lower-limb kinematic and kinetic prediction in studies with EMG and addiction data as input.

Different combinations of muscle activity were used in the studies to predict movement. Figure 6 depicts the most used muscles across these studies were GT and TA. Conclusions about which muscles were relevant for each type of output prediction cannot be drawn currently, due to the low number of papers. Two papers predicted ankle torque [39,40], two papers predicted joint moments [42,43]; Hahn et al. [42] predicted hip, knee, and ankle moments and Zhu et al. [43] predicted only knee moment. Chong et al. [41] predicted knee and hip angle.

Prediction tools again varied, although all could be classified as neural networks; two papers used an artificial neural network (ANN) [40,42], one employed restricted Boltzmann machine (RBM) [41], one combined CNN and LSTM [39], and the final one [43] implemented an Elman neural network. The mechanisms evaluating the performance of the prediction tools were again heterogeneous, limiting direct comparison between studies. While EMG data alone were used to predicted joint angles, EMG combined with additional data were used to predict torques, moments, and/or joint angles. Two papers [39,42] used EMG, angular velocity, and anthropometrics as input to calculate joint moments. Two papers [40,43] used EMG and angle joints as input to calculate torque and moment joints. One paper [41] used EMG, acceleration, and force sensing resistors (FSRs) as an input to calculate joint angles, where the prediction system is seen to perform better than in the previous studies [39,40,42,43], as can be shown in Table 5.

Chong et al. [41] evaluated how much each sensor that measured acceleration (ACC), FSR, and EMG contributed to the prediction of hip and knee angles during natural and controlled speed gait. The results indicate that the least relevant information is ACC in the y direction on the left GT. FSRs on the right and left heel were the most important sensors for prediction. Zhang et al. [40] utilised two models to predict ankle joint torque during natural and controlled gait speed, using an input of ankle angle and calf muscle activity: an EMG-driven neuromusculoskeletal (NMS) model and an ANN. The findings attest that the ANN model predicts torque significantly better (lower RMSE of torque prediction) than NMS, notably during slow walking movement. Moreover, the NMS model and the ANN agreed better with measured torque during gait than in isokinetic ankle movements.

The results indicate the 64 sensors employed to measure ACCs, FSRs, and EMGs could be reduced to 19 sensors while maintaining good prediction performance of angle joints. Hahn et al. [42] intended to predict hip, knee, and ankle moments during natural gait speed using a three-layer forward ANN model. The performance and prediction accuracy of moments were similar across all joints and had acceptable low error values in almost all peak moment comparisons. However, EMGs did not impact the prediction accuracy, with kinematic and demographic data as the input the model achieving accurate joint moment patterns. Moreira et al. [39] predicted the ankle torque of subjects during controlled speed gait with CNNs and LSTMs fed with 11 different variables (Table 5). The results indicate that (i) CNN appears to better estimate ankle joint torque trajectories compared to LSTM, (ii) prediction time using LSTM is higher than CNN by 4.18 ms, (iii) the performance of CNN with or without the EMGs of TA and GT is statistically unchanged. Lastly, Zhu et al. [43] predicted the ankle moment of five healthy subjects walking overground with Elman neural network fed with EMG signals from BF, VL, GT, SEM, thigh angle, and shank angle.

## 4. Discussion

Research to date on gait has focused on lower-limb muscle, brain activity, and kinematic movement as separate entities [44,45,46] examining different conditions or different populations [20,21,22]. Results indicate that kinematics, EMGs, and EEGs are all susceptible to subjects’ physiology and their environment. This systematic review focused on prediction of gait (as measured by lower-limb kinematics and kinetics (joint moment and torque).

Our objective was to systematically gather all data relating to neurological signals that can accurately predict lower-limb kinematics and kinetics for future BCI rehabilitation purposes. We therefore excluded studies where subjects did not perform overground walking in a straight line or used devices such as prostheses, canes, and experimental set-ups that change the natural gait strategy, such as placing weights on the lower-limb extremities or walking on treadmill [47,48]. Descriptive studies, as well as classification- based BCI technologies detecting events related to movement volition [49] or recognizing different movements from several options [50,51], were also excluded. This narrow lens by which to study brain and lower-limb muscle activity that can predict overground stepping and walking resulted in 15 papers out of 6,455 potential papers identified excluding research examining and comparing EEGs, EMGs, and kinematics [52,53,54], applications including pathology recognition [55,56], and identification of different gait phases [57,58]. Classification-based BCI models do not require active participation during the movement execution, thereby limiting positive neuroplastic changes and may, in part, explain the lack of proven long-term benefits associated with BCI interventions employed in rehabilitation [12], despite superior short-term performance to more conventional rehabilitation treatments [59]. Little is known about the neural control of lower-limb movements [60], nevertheless, studies have confirmed the feasibility of decoding linear and angular lower-limb kinematics and kinetics [27,28,61,62] under different conditions. Currently, prediction systems can accurately reconstruct the shape of kinematic signals but fail to estimate the peak amplitudes in real time. These are important considerations to allow the safe implementation of BCI applications and to facilitate, for example, positive neuroplasticity. Reviews related to predicting lower-limb kinematics and/or kinetics to date have focused on signal processing, feature extraction, prediction system employed, execution time, and accuracy of prediction [23,25,63]. To our knowledge, this is the first research paper that systematically gathers the available data in a way that addresses what is currently known about the lower-limb muscle activity and/or brain zones that are most relevant for lower-limb kinematic and kinetic prediction. This systematic review gives an early indication of the EEG electrode locations and EMG targets that may have the most relevant for the prediction of lower-limb kinematics and kinetics as well as the prediction systems most employed and that give the highest accuracy. In the following sections, we discuss the scientific gaps identified from the review and how our synthesized findings about EEGs, EMGs, and prediction tools add to the current scientific literature.

### 4.1. Scientific Gaps

Two reviews [64,65] related to gait prediction reported that most identified studies have used a combination of kinematics and lower-limb muscle activity, with limited studies combining EMGs, EEGs, and kinematics such as Tortora et al.’s study [66]. The findings from our current review are consistent with this, highlighting a lack of attention in the literature to muti-source EEGs, EMGs, and kinematics combination in prediction systems, as no study was identified. From the 15 papers identified that predict lower-limb kinematics/kinetics, only 2 papers utilised EEG data, 8 employed EMGs, and 5 used EMGs with complementary data.

It is also worth noting that different studies [39,67] comparing kinematics and EMG activity of lower-limb muscles during different walking speeds identified intra- and inter-variability in lower-limb biomechanics across subjects.

While most studies included in this review allowed participants to walk at their natural speed, as was reported in other reviews [51,68], in two studies individuals were asked to walk at different speeds [32,39] and in two studies to walk at their natural speed and at other controlled speeds [40,41]. Studies identified a clear decrease in accuracy prediction when subjects walk at a slower speed [39,40,69], indicating that accuracy during protocols employing the same prediction system but different speed gait should not be compared [70].

A recent review focused on decoding the dynamic movement of level-ground walking, stair ascending and descending, ramp ascending and descending in comparison to static movement identified similar limitations in the contemporary literature [51]. The review did not include stepping activity, as we did in the current review. Benefits of studying stepping as an alternative to walking include artifact reduction in EEG and EMG signals [71,72], in comparison to overground gait and a decrease in data acquisition volume. To date, little has been studied in relation to stepping. This systematic review identified only one paper examining stepping activity, implying further studies require focus on this movement.

### 4.2. EEG

It is well established that the primary sensorimotor cortex in subjects with and without neurological disorders contains information about lower-limb kinematics [62,73]. This systematic review confirms EEG electrodes Cz, C3, and C4 contributed to the most accurate predictions of linear and angular kinematics using central neural biosignals [62]. Results from the papers included also show that activation of the brain region related to the dominant lower limb achieves a better prediction performance than in the non-dominant lower limb [28], an interesting observation that requires further research. 

### 4.3. EMG

Studies identified in this review that used EMG signals in neural networks primarily predicted angle joints (69%), while 31% of studies predicted torque or moment joints. A systematic review by Kolaghassi et al. [23], focussed on continuous variable prediction only, identified a similar bias in the scientific literature where 72% of studies predicted angle joints and only 28% predicted torque and moment joints. The majority of studies employing EMG only to predict movement measured lower-limb muscle groups of RF and VL, two muscles that are highly active during gait [74,75]. Both muscles extend the knee joint.

Posterior calf muscles influencing ankle and foot movements were less utilised in predictive systems despite also being active in the gait cycle. Further studies are needed to identify if kinematic predictions without the use of posterior calf muscles as an input to the prediction system are more efficient than using both thigh and calf muscles. On the other hand, studies that fed future deep learning systems with EMG and included other demographic and/or physiological variables as input primarily used both anterior and posterior calf muscle groups (GT and TA). The limited number of studies in this area, in addition to heterogeneity across the papers, limits the conclusions to be drawn about which muscles give the best prediction outcomes. However, what is clear from the findings in this review is that the overall performance of prediction systems is improved when EMG data are supplemented with additional data, although whether the non-EMG data are complimentary or contain more relevant kinematic information requires further elucidation.

### 4.4. Prediction Tools

Predictive systems applied to gait have previously been categorised into four main groups: neural networks, regression methods, statistical models, and decision tree algorithms, with neural networks being the most predominant in the literature [25]. This systematic review found neural networks to be the most used predictive system in lower-limb kinematic prediction, further identifying LSTM, and CNN as the best neural networks tools for systems fed only with EMG data. The best prediction tool across all studies and for systems fed with EEGs or EMGs, or EMGs with other complementary data was not possible to identify due to the reduced number of papers, and the diversity in the systems utilised.

Kalman filter, a statistical model, and Random Forest, a tree algorithm, were identified as used in some of the included studies, while no regression methods were reported. It is worth noting that the mathematical tools most used across all studies to evaluate the performance of the prediction systems were RMSE and CC. However, these mathematical tools give little information about how the neural network generated the relationship between output and input (and if it is logical). In addition, neither tool provides insight about the speed at which the models computed the output, or which information is captured in each layer of the neural networks. It is important to acknowledge that while these methods are suitable to quickly evaluate which tools make the smallest error across prediction systems such as carried out by Kumar [76], the need to design a protocol to evaluate the speed, robustness, and the relevance of the information that neural networks built in each of its layers is also required to aid in their potential feasibility in responsive rehabilitative robotics.

Another aspect warranting future consideration is the optimal information (e.g., the number of electrodes) needed to feed an accurate prediction system. Neural networks can be fed with a large amount of data that do not necessarily translate into high accuracy as the neural network will absorb into its structure attributes of input signals that are not necessarily related to the output. This makes the neural network more susceptible to noise, and a phenomenon called overfitting [77]. When employing EMG, ACC, and FSR input, Chong et al. [41] demonstrated that predictive performance did not increase significantly after the 20th electrode. A similar conclusion was drawn when employing EEG input [73] where temporal or frontal EEG electrodes were noted to make no contribution to the reconstruction of lower-limb movement trajectory. The lower-limb muscle groups and EEG electrodes spotlighted in this systematic review constitute only the starting point towards the identification of a minimum number of electrodes required to predict shape and amplitude of the lower-limb kinematics. More research is required.

It was interesting to note from this review that the predominant focus for both EMG and EEG data was movement prediction. Predictive systems in BCI could utilise these data with alternative roles whereby EEG and/or other physiological data could be used as the predictive input and EMG used as an assessment tool that can define how much effort the subject or the motor of the exoskeleton needs to perform to maintain the planned physical activity during the movement task. This would help ensure that the planned movement task is ultimately completed and allow exoskeleton assistance, for example, to be tailored to provide only the minimal assistance required during rehabilitation.

### 4.5. Limitations

This study, identifying the lower-limb muscles and brain electrodes most employed in neural networks and other predictive systems and the accuracy of their performance, must be considered in light of a number of limitations. The narrow focus taken for overground walking excluded a sizable body of scientific work in other gait contexts such as treadmill training. However, the physiological basis for treadmill walking does differ to that of overground walking and would introduce a confounding factor. In addition, studies employing only kinetic variables such as GRF were excluded. While GRF was previously identified as the variable most used in gait predictive systems [25], we excluded these studies as GRF (i) has no direct correlation with lower-limb articulation, (ii) is associated with inherent difficulty in measuring for long periods of time or across different environments, and (iii) the presence of force plates required to measure GRF alters the floor underfoot and potentially the walking pattern [65].

Overall, the quality of the scientific papers identified, when reviewed using a risk-of- bias tool developed for quantitative, health-related studies, was of low quality. Only one paper was rated as having moderate quality. As the literature in this area comes predominantly from the field of engineering, it raises the need for researchers to target and design clearer procedures for participant recruitment, in line with more clinically focused research. A similar issue was reported in a previous review in robotics in stroke noting that none of the papers reviewed provided a clear rationale for their selection of stroke participants, and provided limited details on stroke pathology, stroke laterality, and stroke severity levels were documented [51,78].

In the upper limb, a systematic review of EMG-based motor intention prediction of continuous upper limb motion published in 2019 [79] demonstrated prediction of both kinematics and kinetics is possible and precise using EMG data only. However, identified studies were conducted offline with no knowledge of the predictive performance online. Similar limitations in lower-limb prediction systems employing EEGs, or EMGs, are reflected in our systematic review where the evaluation of all prediction systems (primarily EMGs) was performed offline.

Finally, the results identified in this systematic review may limit broad applicability. The population reported across the included studies is limited to individuals under 50 years old, mostly to healthy individuals and primarily from data captured without exoskeleton use. Conclusions drawn from the findings of this review lack biosignal information related to human machine interaction and provide limited data related to central neurological disorders, to whom BCI technology will be most applicable.

## 5. Conclusions

This systematic review highlights that the primary motor electrodes (Cz, C3, C4), quadriceps femoris muscles and posterior calf muscles (RF, VL, GT, and TA), and neural networks (CNN and LSTM) are the most commonly registered and used signals and tools in lower-limb motion prediction and demonstrate the highest performance in the prediction of lower-limb kinematics and kinetics based on the contemporary research in this filed. Moreover, several gaps have been identified that are critical for furthering development of prediction system technology. These include the absence of (i) predictions combining EEGs and EMGs, (ii) standardized statistical tools to evaluate the prediction tools, and (iii) targeted populations with central neurological disorders and those over 50 years of age.

## Figures and Tables

**Table 1:** Synonym of the keywords used in the databases search.

	Keyword	Synonyms
#1	EEG	(electroencephalog* OR EEG OR “brain activity” OR “brain electric? Activity” OR “brain wave*” OR “brainwave*” OR “e.e.g” OR “electr* encephalogram*” OR cEEG OR “EEG-based BCI” OR “EEG-BCI” OR “EEG-based brain-computer interface” OR “EEG-brain computer interface”)
#2	EMG	(electromyogra* OR EMG OR sEMG OR “e.m.g” OR “electr* myogram”)
#3	BCI	(“brain-machine interfac*” OR “brain machine interfac*” OR “brain-computer interfac*” OR “brain computer interface” OR “brain computing interface*” OR “mind-machine interface” OR “mind machine interface” OR “cerebral-computer interfac*” OR “cerebellum-machine interfac*” OR “direct neural interface*” OR BCI OR BCIs OR “neural interface system*” OR “neural-interface system” OR “BCI-controlled neuroprosthetic” OR “human machine interface” OR “human-machine interface” OR HMI OR HMIs OT HCI OR HCIs OR “robotic walking device*” OR “restorative robotic device*”)
#4	ERS/ERD/ERSP	(“ERD/S” OR “ERD/ERS” OR ERD OR “event-related desynchroni$ation” OR “event-related synchroni$ation” OR ERS OR ERSP OR “event-related spectral perturbation” OR “event-related spectral power” OR “event-related slow potential” OR “event related spectral perturbation” OR “event related spectral power” OR “event related slow potential” OR “corticomuscular coherence” OR CMC OR “evoked action potential” OR “evoked discharge” OR “evoked nerve action potential” OR “evoked nerve response” OR “evoked potential” OR “evoked potentials” OR “cortical synchroni$ation*” OR “cortical phase synchroni$ation” OR “cortical desynchroni$ation*” OR “cortical phase desynchroni$ation” OR “cortical phase desynchroni$ation*” OR “phase desynchronization*” OR “event related potential*” OR “event-related potential”)
#5	Stepping	(step* OR walk* OR gait* OR stride OR ambulat* OR stance OR swing OR “toe-off” OR “heel-strike” OR mobili$at*)
#6	Kinematics	(kinematic* OR biomechanic* OR dynamic* OR “motion analys*” OR “movement analys*” OR “ kinesiology” OR “motion tracking system” OR “motion-tracking biomechanical function analysis system” OR “biomechanical phenomena”)
#7	MTP	(“motion trajectory prediction” OR “motion analysis system” OR “motion analysis device” OR “motion capture system” OR “monitor capture device”)
#8	Locomotion	(locomot* OR “motor behavior” OR “motor behaviour”)

**Table 2:** Summary of conclusions gathered from the analysis of results.

	EEGs	EMGs	EMGs + Data
**COMMENTS**	There are relevant data on the time domain of EEGs for the prediction of hip and knee torques during gait in healthy young adults. There are relevant data in the frequency domain of EEGs for prediction of hip, knee, and ankle angles during robotic stepping in adult stroke subjects. To date, electrodes yielding most information for the prediction of torque kinematics from healthy subjects are centrally located in the brain.	The tools identified which best predicted kinematics/kinetics were fed with data from only one lower-limb side. Lower-limb EMG data most used in kinematic/kinetic prediction was derived from thigh muscles (RF and VL) acting on the knee joint. The parameter most predicted across studies was the knee angle. The prediction tools most employed and moreover identified to have the highest accuracies were neural networks: CNN and/or LSTM. The statistical tools most used across studies to evaluate neural networks performance were RMSE and CC.	Lower-limb EMG activity most utilised in combined kinematic and kinetic predictions was derived from calf muscles (GT and TA) working around the ankle joint. Muscles that make the strongest contribution to kinetic and kinematic prediction cannot be currently defined due to the heterogeneity in outputs reported and statistical tools utilised. Ankle and knee kinematics and kinetics are equally predicted. No conclusion about which prediction tools are most used or useful can be drawn due to also the heterogeneity of prediction and statistical tools reported across studies. No study using EMGs with additional data predicted lower-limb kinematics in subjects using an exoskeleton.

**Table 3:** Descriptive characteristics of subjects, protocol, signal processing, and output of studies with only EEG input.

Authors	Participants	Protocol	Data		Pre-Processing		Prediction	Accuracy	
			Input	Output	Input	Output			
Contreras-Vidal et al., 2018 [27]	1F/5M Chronic poststroke hemiparesis (H: 160–192 cm, A: 40–68 yrs, W: 62–99 kg)	Walking with exoskeleton Natural speed	EEGs (64 channels, 10–20 system)	Hip, knee, and ankle angles	Artificial subspace reconstruction Peripheral channel removal Detrendation Common average referencing Down-sampling to 100 Hz Butterworth band-pass filter (0.1–3 Hz, 4th) Standardization	Low-pass filter (3 Hz)	10th order unscented Kalman filter	RMSE ^1^	
Mercado et al., 2021 [28]	8F/12M Healthy subjects (A: 21–23)	Step forward, up, and back Natural speed	EEG (19 channels, 10–20 system)	Hip and knee torques	Notch filter at 60 Hz SOBI-RO K-nearest neighbours	Conversion of RGB video to BW	Multi-layer perceptron	RMSE (°): Right hip Left hip Right knee Left knee	0.0023 0.0018 0.0095 0.0051

^1^ RMSE values were summarised in plots. For further details, consult Contrerass-Vidal et al.’s study [22].

**Table 4:** Descriptive characteristics of subjects, protocol, signal processing, and output of studies with only an EMG input.

Authors	Participants	Protocol	Data		Pre-Processing		Prediction	Accuracy		
			Input	Output	Input	Output				
Brantley et al., 2017 [31]	1F/5M Healthy subjects	Walking, stair descent/ascent, ramp descent/ascent Natural speed	EMG of VL, RF, BF, SEM	Ankle and knee angles	Normalization Butterworth band-pass filter (30–350 Hz, 4th) Rectification Butterworth low-pass filter (6 Hz, 4th)	Butterworth low-pass filter (6 Hz, 4th)	Unscented Kalman filter	Pearson’s correlation coefficient: 0.643		
Chen et al., 2017 [32]	0F/6M Healthy subjects (H: 170.6 ± 3.6, A: 26 ± 2.2 yrs, W: 62.6 ± 3.7 kg)	Walking Controlled speed	EMG right limb of BF, SEM, VM, VL, RF, SR, MG, LG, TA, SOL	Hip, knee and ankle angles	Notch filter 50 Hz Zero-lag fourth-order recursive Butterworth filter with 20 Hz Full-wave rectification Sub-sampling at 100 Hz Butterworth low-pass filter (4 Hz)		BP Neural network with: DBN and PCA.	RMSE(°) ^1^:		
									DBN	PCA
								Hip	3.58 ± 0.67	6.22 ± 1.67
								Knee	3.96 ± 0.69	8.11 ± 2.02
								Ankle	2.45 ± 0.57	4.65 ± 1.32
Cheron et al., 2003 [33]	5F/4M Healthy subjects (A: 35 + 6 yrs)	Walking Natural speed	EMG left limb of RF, VL, BF, TA, GL, SOL	Hip, knee and ankle angles, angular velocity and angular acceleration	Band-pass filter (5–2000 Hz) Full-wave rectification Smoothing with a third-order averaging filter		DRNN	Consult reference		
Gautam et al., 2020 [34]	0F/11M Healthy subjects	Walking, sitting and standing Natural speed	EMG left limb of VM, SEM, BF, RF	Knee angle	Band-pass filter (20–460 Hz)	Empirical Mode Iterative Algorithm (EIA)	CNN + LSTM	MAE ± SDMAE (%): 8.1 ± 1.2		
Jia et al., 2021 [35]	0F/4M Healthy subjects (H: 172.1 ± 5.8 cm, A: 23.6 ± 1.4 yrs, W: 65.2 ± 7.5 kg)	Walking Natural speed	EMG left limb of RF, VL, GM	Knee angle	Full-wave rectification Butterworth low-pass filter (30 Hz, 6th)		Traditional LSTM Traditional RNN Adopted LSTM	RMSE ± RMSE (°) employing: Traditional LSTM 1.5 ± 0.098 Traditional RNN 2.523 ± 0.373 Adopted LSTM 0.464 ± 0.096 Correlation Coefficient ^1^: Traditional LSTM 0.984 ± 0.00219 Traditional RNN 0.963 ± 0.01223 Adopted LSTM 0.999 ± 0.00001		
Li et al., 2019 [36]	0F/6M Healthy subjects (H: 181 ± 3.8 cm, A: 24.2 ± 1.6 yrs, W: 72.5 ± 6.9 kg)	Walking Natural speed	Unilateral EMG of VL, RF, VM, GM, GL	Knee angle	Butterworth band-pass filter (10–500 Hz, 4th) Rectification Low-pass filter (6 Hz, 2nd) Resample 100 Hz		Principal Component with: Backpropagation Random Forest	RMSE (°): Backpropagation 13 Random Forest 5		
Liu et al., 2019 [37]	0F/3M Healthy subjects (H: 177.6 ± 2.5 cm, A: 22 ± 1 yrs, W: 70.6 ± 1.9 kg, BMI: 22.5 ± 0.4 kg/cm)	Walking Natural speed	EMG left limb of VL, RF, VM, BF, SEM, MG	Knee angle	Butterworth band-pass filter (20–460 Hz) Butterworth notch filter at 50 Hz Full-wave rectification Normalization	Butterworth low-pass filter (6 Hz, 4th)	BPNN Original data-based CNN Feature-based CNN	RMSE (°): BPNN 9.15 Original data-based CNN 10.57 Feature data-based CNN 5.88 Coefficient of correlation: BPNN 0.96 Original data-based CNN 0.93 Feature data-based CNN 0.98		
Wang et al., 2015 [38]	5 Subjects (H: 173 cm, A: 21 yrs, W: 67 kg)	Walking Natural speed	EMG left limb of VL, TA, GM	Knee angle			GA-GRNN	RMSE ± MPE ^2^(°): 0.6406 ± 0.9331 Coefficient of correlation: 0.9983		

^1^ Performance averaged along the different speeds.; ^2^ MPE stands for Maximum Permissible Error.

**Table 5:** Descriptive characteristics of subjects, protocol, signal processing, and output of studies with EMG input and additional input data.

Authors	Participants	Protocol	Data		Pre-Processing		Prediction	Accuracy
			Input	Output	Input	Output		
**Zhang et al., 2021** [40]	4F/6M Healthy subjects (H: 175.06 ± 8.45 cm, A: 26 ± 2.86 yrs, W: 70.36 ± 11.49 kg)	Walking Natural and controlled speed	EMG right limb of SOL, TA, GM Ankle joint	Ankle torque	EMG: Band-pass filter (20–500 Hz) Rectification Low-pass filter (6 Hz) Normalisation	Butterworth low-pass filter (6 Hz, 4th order) GRF: Low-pass filter	EMG-driven NMS model ANN model	RMSE (Nm/Kg): Fast walking speed 0.06 ± 0.03 Slow walking speed 0.01 ± 0.01 Self-selected walking speed 0.08 ± 0.06
**Chong et al., 2017** [41]	0F/4M Healthy subjects (H: 177.9 ± 3.18 cm, A: 26.75 ± 4.32 yrs, W: 81.5 ± 8.44 kg)	Walking Natural and controlled speed	EMG of RF, VM, TA, GM, BF, GT, SOL ACC FSR	Knee and hip angles			Boltzmann machine (RBM)	MSE ± STD(MSE) (°): Right knee 0.1768 ± 0.3736 Right hip 0.1444 ± 0.3628 Left knee 0.1680 ± 0.3592 Left hip 0.1756 ± 0.4040
**Hahn et al., 2008** [42]	12F/7M Healthy subjects (H: 173 ± 0.08 cm, A: 22.3 ± 1.6 yrs, W: 72 ± 13.3 kg)	Walking Natural speed	EMG of GMAX, GMED, BF, RF, VL, TA, MG Demographics Anthropometrics Joints angles, acceleration, and angular velocity	Hip, knee and ankle moments	EMG: Bandwidth filter (10–1000 Hz) Full-wave rectification Envelopment with a low-pass filter (5 Hz, 4th) Magnitude-normalisation to the maximum value of the trial Joints coordinates: Woltring filter		Three-layer feedforward ANN structure	Coefficient of determination: Hip 0.95 Knee 0.94 Ankle 0.99
**Moreira et al., 2021** [39]	7F/6M Healthy subjects (H: 168 + 1.2, A: 24.2 + 1.85 yrs, W: 65.2 + 10.3 kg)	Walking Controlled speed	EMG of TA, GAL Ankle angle Angular velocities Angular accelerations Walking speed Body mass, and height Foot and shank length Gender Age	Ankle torque	EMG: Butterworth band-pass filter (20–450 Hz) Enveloping with Root Mean Square Kinematics: Low-pass filter (6 Hz)		LSTM CNN	NRMSE: LSTM 0.48 CNN 0.72 Coefficient of correlation: LSTM 0.73 CNN 0.92
**Zhu et al., 2019** [43]	0F/5M Healthy subjects (H: 173 ± 0.05, A: 24 ± 1.5 yrs, W: 60.5 ± 4.6 kg)	Walking Natural speed	EMG of BF, VL, GA, SE Thigh angle and shank angle	Knee joint moment	Butterworth band-pass filter (8–500 Hz, 4th) Notch filter 50 Hz Wave rectifier		Elman neural network	NRMSE: 0.116 Coefficient of correlation: 0.979

## Data Availability

All data collected have been displayed in the publication.
